# Increased maternal hCG concentrations in early *in vitro* pregnancy with elevated basal FSH

**DOI:** 10.1371/journal.pone.0203610

**Published:** 2018-09-10

**Authors:** Xiao Han, Baoli Yin, Shengli Lin, Qian Wang, Ni Su, Cuilian Zhang

**Affiliations:** 1 Reproductive Medical Center, People’s Hospital of Zhengzhou University, Zhengzhou, Henan Province, People’s Republic of China; 2 Academy of Medical Science, Zhengzhou University, Zhengzhou, Henan Province, People’s Republic of China; 3 Reproductive Medical Center, Department of Obstetrics and Gynecology, Peking University Third Hospital, Beijing, People’s Republic of China; University of Crete, GREECE

## Abstract

**Objective:**

To investigate factors that influence maternal hCG concentration in early pregnancy and the relationship between hCG concentration in early pregnancy and basal FSH (bFSH) level.

**Design:**

Retrospective cohort study.

**Setting:**

Reproductive medical center.

**Patient(s):**

In total, 482 women aged 22 to 38 years with elevated basal FSH (> 10 IU/L) and who experienced a single live birth after *in vitro* fertilization-embryo transfer were selected. These 482 women were age-matched with an equal number of women with normal basal FSH (≤10 IU/L) who also experienced a single live birth.

**Intervention(s):**

None.

**Main Outcome Measure(s):**

HCG concentration.

**Result(s):**

The hCG concentrations on Day 14 and Day 21 were 560.46 (363.63–842.52) IU/L and 9862.00 (6512.25–14029.50) IU/L, respectively, in the elevated bFSH group, and these values were significantly increased compared with the normal bFSH group. After adjusting for confounding factors, the concentrations of maternal hCG on Day 14 and Day 21 were significantly associated with basal FSH. In addition, crude linear regression analysis demonstrated that hCG concentrations increased as the basal serum levels of FSH increased.

**Conclusion(s):**

Elevated basal FSH has implications for the interpretation of hCG concentrations in early pregnancy after *in vitro* fertilization-embryo transfer (IVF-ET) that led to a single live birth.

## Introduction

Human chorionic gonadotrophin (hCG), a hormone produced by placental trophoblast cells, can reflect trophoblastic mass and is the earliest marker available to evaluate pregnancy progression [1]. Shortly after implantation, hCG levels dynamically increase and can be detected as early as 6–8 days in maternal serum [2]. In addition to serving as an indicator to evaluate pregnancy progression, hCG in early pregnancy exhibits several other physiological functions for embryo development. In addition to its primary role in maintaining the corpus luteum, hCG is also crucial in trophoblast proliferation and angiogenesis, thus facilitating placental development [3]. Several studies have revealed the association between low early maternal hCG level and risk of compromised pregnancy outcome. Asvold *et al*. found that lower maternal serum hCG on day 12 after embryo transfer was an independent predictor of severe pre-eclampsia [4]. Morse *et al*. reported that an increase in hCG very early in the first trimester was associated with reduced newborn birth weight [5]. All these findings indicate that hCG is a crucial indicator of pregnancy outcome.

The responses to ovarian stimulation varied greatly among individuals, even individuals of the same age during in vitro fertilization-embryo transfer (IVF-ET). Decreased ovarian reserve (DOR) is used to characterize this variation and is defined as women of reproductive age and regular menses who respond poorly to ovarian stimulation compared with others of the same age [6]. A unified definition for decreased ovarian reserve (DOR) is not currently available. Most of the studies demonstrate that FSH>10 IU/L could reflect DOR. In addition, age, AFC and AMH could also reflect DOR; however, the cut-off values remain undefined[7,8]. FSH is secreted by the anterior pituitary gland and contributes to follicle development, and an increase in FSH could result from the reduced quantity and quality of antral follicles. Reduced antral follicle count (AFC) results in decreased estradiol (E2) and inhibin b. This process can weaken the negative feedback of follicle-stimulating hormone (FSH) and subsequently reduce FSH levels. Therefore, FSH could be regarded as a marker to evaluate ovarian reserve [9]. Recent studies examined the factors that influence maternal hCG concentrations in early IVF pregnancy [10]. In the present study, we sought to investigate whether basal FSH levels affect early maternal hCG concentrations after IVF-ET. To answer this question, we studied the relationship between basal FSH and hCG concentrations on Day 14 and Day 21 after embryo transfer, which to a certain extent could reflect the relationship between ovarian reserve and hCG concentrations in early pregnancy.

## Materials and methods

### Patients

Our retrospective study was reviewed and approved by the Ethics Committee of People’s Hospital of Zhengzhou University. Patients did not sign informed consent because our study meets the criteria of exemption from informed consent. Thus, the Ethics Committee did not require informed consent to be obtained. All fresh embryo transfer cycles that led to a single live birth were included. Clinical pregnancy was diagnosed based on the detection of a single gestational sac with a fetal heartbeat at 7 weeks of gestation.

Early follicular phase serum FSH levels were determined with a commercial immunoassay system (Siemens, Immulite 2000 FSH). In total, 482 patients with increased basal serum FSH (>10 IU/L) were included in group A and were age-matched with an equal number of patients with normal basal FSH (≤10 IU/L) as group B. Patients were excluded from this study if they received a preimplantation genetic diagnosis (PGD) or required an oocyte donation.

Patients participating in fresh embryo transfer cycles underwent controlled ovarian hyperstimulation with a gonadotropin-releasing hormone (GnRH) agonist or GnRH antagonist protocol as described previously [11]. Ovarian follicle development was monitored based on serum estradiol (E2) levels and transvaginal ultrasonographic measurements. When at least one follicle reached a diameter of 18 mm and the E2 level exceeded 500 pg/ml, 10,000 units of urinary hCG (Serono, Aubonne, Switzerland) were administered before ultrasonography-guided oocyte retrieval. Regular luteal support was administered with 60-mg progesterone intramuscular injection or vaginal progesterone (8% Crinone vaginal gel, Merck-Serono) daily.

### Laboratory protocols

IVF and intra-cytoplasmic sperm injection (ICSI) were performed according to routine laboratory insemination procedures on the day of oocyte retrieval. The presence of two pronuclei was observed 17 ± 1 h after insemination or injection, and the zygotes were then cultured in 25 μl pre-equilibrated cleavage medium droplets. The embryos were cultured in incubators at 37°C under 5% or 6% CO_2_. The morphology of embryos was evaluated 68 ± 1 h after insemination with respect to cell number, fragmentation, and symmetry. The total number of embryos transferred was determined based on patient age, the number of previous IVF cycles, and embryo quality. The maternal hCG concentrations on Day 14 and Day 21 after embryo transfer were examined using Beckman DxI800 immunoassay systems.

### Statistical analysis

Statistical analyses were conducted using SPSS software (version 16.0). The results for continuous data are reported as the mean ± standard deviation. Categorical variables are reported as frequencies (%) and were evaluated using chi-square tests. Associations between basal FSH level and maternal hCG concentrations were analyzed using linear regression. Multiple linear regression analysis was used to assess the association between basal FSH level and maternal hCG concentrations after adjusting for maternal age, maternal BMI, duration of subfertility, subfertility type, fertilization method (IVF or ICSI), gonadotrophin dose, number of oocytes retrieved and number of transferred embryos.

## Results

The median hCG concentration was 529.02 IU/L on Day 14 and 9188.00 IU/L on Day 21 after embryo transfer. The patient characteristics for groups A and B are presented in Table 1. BMI, duration of subfertility, the number of oocytes retrieved and the number of embryos transferred were reduced in group A compared with group B. In addition, FSH serum levels, the dose of gonadotrophin, and hCG concentrations on Day 14 and Day 21 after embryo transfer were increased in group A compared with group B. Other characteristics, including age, primary infertility rate and rate of cycles with ICSI, were similar between the two groups.

**Table 1:** Maternal hCG concentrations on Days 14 and 21 in age-matched patients according to ovarian reserve status.

Characteristics	Group A (n = 482)	Group B (n = 482)	*P*-value
Age (years)	32.37 ± 3.13	31.97 ± 3.44	0.593
Body mass index (kg/m^2^)	21.55 ± 2.97	22.37 ± 2.94	< 0.001*
Duration of subfertility (years)	4.67 ± 3.14	5.13 ± 3.38	0.027*
Primary subfertility	272 (56.43%)	261 (54.15%)	0.476
Secondary infertility	210 (43.57%)	221 (45.85%)	0.476
FSH (IU/L)	11.60 (10.60–12.80)	6.70 (5.40–7.89)	< 0.001*
Cycles with ICSI	198 (41.08%)	191 (39.63%)	0.646
Dose of gonadotrophin (IU)	3804.12 ± 1510.59	2771.23 ± 1321.50	< 0.001*
No. of oocytes retrieved	8.45 ± 4.95	13.28 ± 6.17	< 0.001*
No. of embryos transferred	2.13 ± 0.55	2.27 ± 0.51	< 0.001*
hCG concentrations (IU/L) on day 14	560.46 (363.63–842.52)	514.86 (311.60–713.23)	0.002*
hCG concentrations (IU/L) on day 21	9862.00 (6512.25–14029.50)	8584.00 (5169.50–12157.25)	< 0.001*

Note: Data are presented as numerical values (%); means ± SD, or median (interquartile ranges). Categorical variables were evaluated using chi-square tests.

*P-values indicate statistically significant differences between the groups.

**Fig 1:**
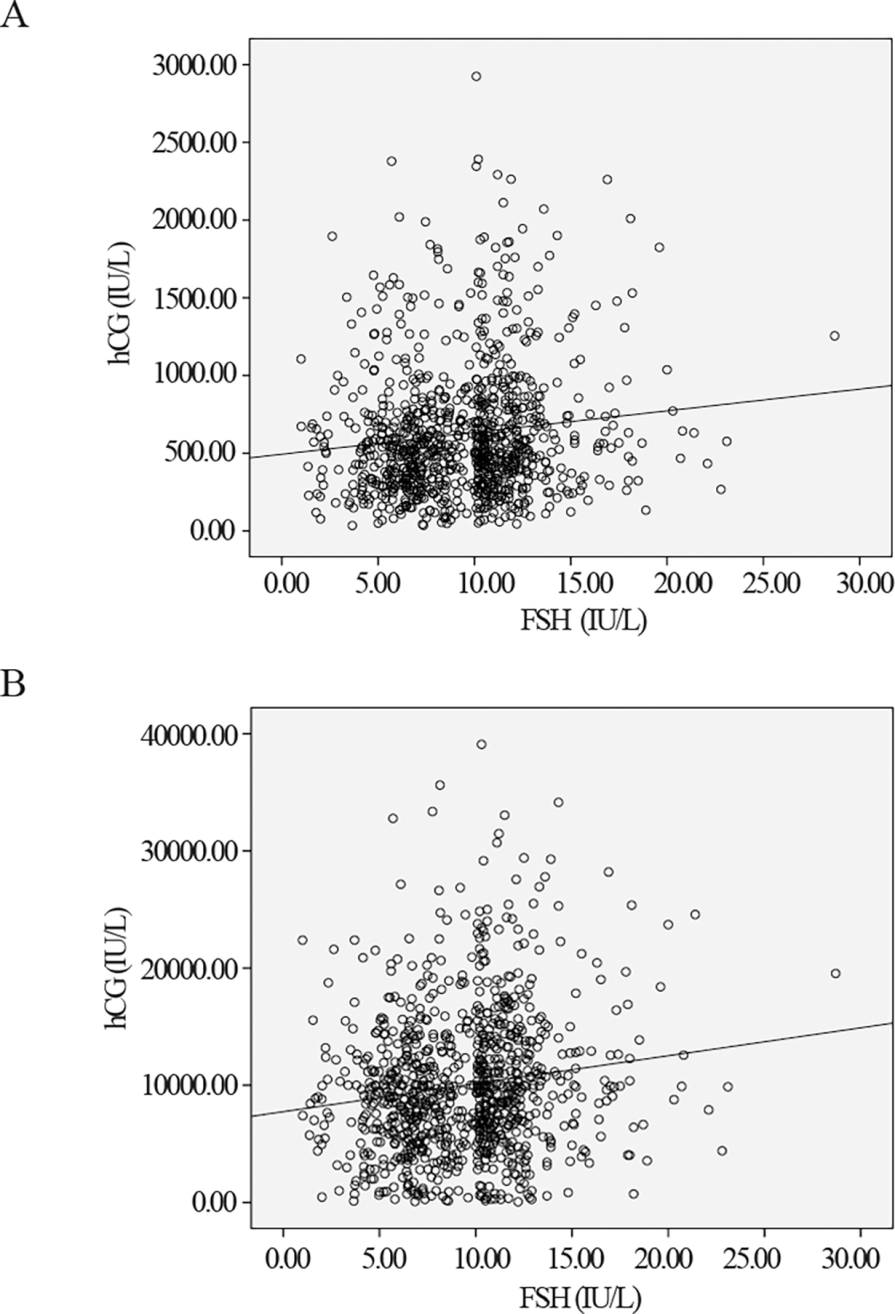
Levels of basal FSH and maternal hCG concentrations. A, The crude association of levels of basal FSH with maternal hCG concentrations 14 days after embryo transfer (linear regression, *P* = 0.014); B, The crude association of levels of basal FSH with maternal hCG concentrations 21 days after embryo transfer (linear regression, *P* = 0.02).

After adjusting for confounding factors, basal FSH level was significantly associated with the concentration of maternal hCG on Day 14 (*P* = 0.026, with group B as the reference group, Table 2) and Day 21 (*P* = 0.036, with group B as the reference group, Table 3). Maternal BMI and number of oocytes retrieved were also significantly related to the concentration of maternal hCG. In addition, crude linear regression analysis demonstrated that hCG concentrations increased as basal FSH serum levels increased (Fig 1).

**Table 2:** Differences in factors associated with hCG concentrations on day 14 after *in vitro* fertilization between women with increased bFSH and normal bFSH.

Parameters	β	t	*P*-value
Increased bFSH (versus normal bFSH)	0.081	2.225	0.026*
Maternal age (years)	0.030	0.796	0.426
Maternal BMI (kg/m^2^)	-0.074	-2.261	0.024*
Duration of subfertility (years)	-0.019	-0.541	0.589
Primary subfertility	-0.053	-1.576	0.115
Cycles with ICSI	0.015	0.440	0.660
Dose of gonadotrophin (IU)	-0.068	-1.851	0.064
No. of oocytes retrieved	-0.107	-2.864	0.004*
No. of embryo transferred	-0.026	-0.732	0.464

Note: β is the regression coefficient

*P-values indicate statistically significant differences between the groups

**Table 3:** Differences in factors associated with hCG concentrations on day 21 after *in vitro* fertilization between women with increased bFSH and with normal bFSH.

Parameters	β	t	*P* value
Increased bFSH (versus normal bFSH)	0.076	2.104	0.036*
Maternal age (years)	0.018	0.478	0.633
Maternal BMI (kg/m^2^)	-0.095	-2.914	0.004*
Duration of subfertility (years)	-0.054	-1.555	0.120
Primary subfertility	-0.038	-1.123	0.262
Cycles with ICSI	0.050	1.505	0.133
Dose of gonadotrophin (IU)	-0.028	-0.766	0.444
No. of oocytes retrieved	-0.104	-2.807	0.005*
No. of embryo transferred	-0.042	-1.206	0.228

Note: β is the regression coefficient

*P-values indicate statistically significant differences between the groups.

## Discussion

In the present study of 964 age-matched patients who experienced a single live birth, maternal hCG concentrations were increased in the increased bFSH group compared with the normal bFSH group on Days 14 and 21 after fresh embryo transfers.

Embryonic development plays an important role in pregnancy outcomes. The serum hCG concentration indicates the trophoblastic mass and function, which can be examined in early pregnancy [12]. hCG is a superior predictor of pregnancy outcomes compared with other biochemical markers [13].

Women who underwent IVF-ET were injected with 10,000 units of urinary hCG (Serono, Aubonne, Switzerland) 36 h before oocyte retrieval to induce ovulation. The hCG possesses a half-life of 2.3 days, but exogenous hCG was metabolized during circulation after 14 days following injection and was not detected in the serum [14]. In the present study, serum hCG concentrations were measured on Days 14 and 21 after embryo transfer. Therefore, the exogenous hCG injected for ovulation would be metabolized before measurement and would unlikely affect the hCG levels.

The follicular microenvironment plays an important role in oocyte competence and early embryogenesis. The communication loop between granulosa cells/cumulus cells and oocytes contributes to oocyte development [15]. The oocyte secretes paracrine factors that regulate the proliferation and differentiation of granulosa cells/cumulus cells. In addition, granulosa cells/cumulus cells are responsible for the growth and maturation of oocytes by providing nutrients and growth factors [16]. Recently, Pacella *et al* reported that the tumor suppressor p53 was significantly increased in cumulus cells in women with reduced ovarian reserve [17]. As a transcription factor, p53 regulates cell division and apoptosis [18]. p53 is involved in the reproduction process by activating leukemia inhibitory factor (LIF) transcription and affects blastocyst implantation [19]. In addition, p53 can bind with the *hCGβ* gene and upregulate its expression [20, 21]. Therefore, it is possible that upregulated p53 expression increased maternal hCG concentrations in early pregnancy among women with increased basal FSH levels. Another possible explanation for the increased hCG concentrations among patients with high basal FSH levels exists. High basal FSH levels are related to reduced ovarian reserve to some certain extent. As the quality and quantity of oocytes and embryos decrease, the pregnancy loss rate significantly improves. Therefore, patients who experience a live birth may require more implanted embryos, which can result in increased hCG levels in early pregnancy. This notion may contribute to the phenomenon whereby early pregnancy hCG levels were increased in patients with increased basal FSH levels. However, the underlying mechanisms still require further research efforts.

Multiple linear regression analysis was performed to determine the associations between confounding factors and maternal hCG concentrations. Consistent with our hypothesis, women in the increased bFSH group exhibited significantly increased concentrations of maternal hCG on Day 14 and Day 21 after embryo transfer. In addition, we also found that maternal BMI and the number of oocytes retrieved were negatively associated with hCG concentrations on Day 14 and Day 21. A previous study demonstrated that increased maternal BMI in early pregnancy reduces serum hCG concentrations [22]. Crude linear regression demonstrated that hCG concentrations also decrease during the retrieval of a high number of oocytes during IVF-ET [3]. It is possible that not only maternal BMI and the number of oocytes retrieved but also basal FSH levels have implications for predicting hCG concentrations in early pregnancy.

Anti-Mullerian hormone (AMH) is a glycoprotein hormone secreted by small ovarian follicles and serves as a strong marker that predicts the number and quality of remaining oocytes in the ovary [23]. Our analysis was limited in that serum AMH levels were not acquired during the study period in this patient cohort. In addition, the present study was limited by its retrospective nature. Moreover, a single live birth may result from a single pregnancy or multiple pregnancies; however, the rate of vanishing multiple pregnancies was unknown in both groups. We were unable to determine the relationship between bFSH and implantation rate as well as pregnancy loss rate. Therefore, prospective studies are needed to explore the association between basal FSH levels and hCG concentrations and determine how these factors are related to serum AMH levels as well as pregnancy loss rate.

In conclusion, our study demonstrates that basal FSH is positively associated with maternal hCG concentrations in early pregnancies that resulted in a single live birth. This finding can be partially interpreted based on the rapid changes of early maternal hCG concentrations following IVF-ET. More research should be conducted to understand the underlying molecular mechanisms.

## Abbreviations

ART: assisted reproductive technology
IVF-ET: *in vitro* fertilization-embryo transfer
DOR: decreased ovarian reserve
FSH: Follicle-stimulating hormone
AMH: Mullerian hormone
ICSI: intra-cytoplasmic sperm injection
BMI: body mass index
GnRH: gonadotropin-releasing hormone
